# Functional outcome of nerve transfer for restoration of shoulder and elbow function in upper brachial plexus injury

**DOI:** 10.1186/1749-7221-3-15

**Published:** 2008-05-27

**Authors:** Hari Venkatramani, Praveen Bhardwaj, Sajedur Reza Faruquee, S Raja Sabapathy

**Affiliations:** 1 Department of Plastic, Hand and Reconstructive Microsurgery, Ganga Hospital, Mettupalayam road, Coimbatore, India

## Abstract

**Background:**

Purpose of this study was to evaluate the functional outcome of spinal accessory to suprascapular nerve transfer (XI-SSN) done for restoration of shoulder function and partial transfer of ulnar nerve to the motor branch to the biceps muscle for the recovery of elbow flexion (Oberlin transfer).

**Methods:**

This is a prospective study involving 15 consecutive cases of upper plexus injury seen between January 2004 and December 2005. The average age of patients was 35.6 yrs (15–52 yrs). The injury-surgery interval was between 2–6 months. All underwent XI-SSN and Oberlin nerve transfer. The coaptation was done close to the biceps muscle to ensure early recovery. The average follow up was 15 months (range 12–36 months). The functional outcome was assessed by measuring range of movements and also on the grading scale proposed by Narakas for shoulder function and Waikakul for elbow function.

**Results:**

Good/Excellent results were seen in 13/15 patients with respect to elbow function and 8/15 for shoulder function. The time required for the first sign of clinical reinnervation of biceps was 3 months 9 days (range 1 month 25 days to 4 months) and for the recovery of antigravity elbow flexion was 5 months (range 3 1/2 months to 8 months). 13 had M4 and two M3 power. On evaluating shoulder function 8/15 regained active abduction, five had M3 and three M4 shoulder abduction. The average range of abduction in these eight patients was 66 degrees (range 45–90). Eight had recovered active external rotation, average 44 degrees (range 15–95). The motor recovery of external rotation was M3 in 5 and M4 in 3. 7/15 had no active abduction/external rotation, but they felt that their shoulder was more stable. Comparable results were observed in both below and above 40 age groups and those with injury to surgery interval less than 3 or 3–6 months.

**Conclusion:**

Transfer of ulnar nerve fascicle to the motor branch of biceps close to the muscle consistently results in early and good recovery of elbow flexion. Shoulder abduction and external rotation show modest but useful recovery and about half can be expected to have active movements. Two patients in early fifties also achieved good results and hence this procedure should be offered to this age group also. Surgery done earlier to 6 months gives consistently good results.

## Background

In upper brachial plexus avulsion injuries loss of abduction and external rotation at shoulder and flexion at elbow are the main functional deficits. Spinal accessory nerve has been the most commonly used donor for restoration of shoulder abduction and external rotation with varying results in different centers. [1-9]. On average, the results have been modest [10]. Many donors have been tried for restoration of elbow flexion with overall good results [1-5,11-14]. Oberlin et al [15] described the partial transfer of ulnar nerve to the motor branch of biceps muscle. This procedure has consistently shown good results [16-19]. Since the coaptation is done very near to the muscle and without nerve grafts early recovery is possible. No significant donor deficits have been reported [15-19]. We present our experience with fifteen consecutive cases of upper plexus injury treated by this set of nerve transfers, involving spinal accessory to suprascapular (XI-SSN) and partial transfer of ulnar nerve to the biceps motor branch (UNF-BrBi – Oberlin transfer).

Recent literature [20] and our experience also suggest that additional transfer of median nerve fascicle to the motor branch to brachialis gives even better results. This protocol is followed from December 2005. The results presented in this series are of transfer of only the ulnar nerve fascicles (Oberlin procedure).

## Methods

This was a prospective study of 15 consecutive cases of upper plexus injury with good hand function who presented to us within 6 months of injury during the period of January 2004 and December 2005. The average age of patients was 35.6 yrs (15–52 yrs). There were 14 males and one female. In all the cases mode of injury was road traffic accident. All the patients were right hand dominant and 14 had injury to the dominant side. The surgery was done between 2 and 6 months after the accident. The average follow up was 15 months (range 12 to 36 months). Spinal accessory to suprascapular nerve transfer (XI-SSN) and transfer of ulnar nerve fascicle to the motor branch to biceps (UNF-BrBi – Oberlin transfer) was done in all of them.

A detailed preoperative assessment was done in all the case and documented as the base line. Elbow flexion, shoulder abduction, and external rotation range of motion were 0 degree in all the cases. Deltoid, teres minor, supraspinatus, infraspinatus, biceps, and brachioradialis muscle were all paralyzed and scored M0 on Medical Research Council (MRC) scoring. In two cases triceps scored M0 (case no. 5 and 6), others had M4–M5 score. Trapezius muscle was scored M5 and grip and pinch strength in the hand was normal in all the cases.

With a minimum follow up of 12 months, all of them were evaluated for range of movements at shoulder and elbow; motor power and functional improvement.

### Outcome assessment

Strength of muscle was graded using MRC scoring and range of movements was recorder with Goniometry. The range of elbow flexion was measured as the angle formed between the long axis of the arm and the forearm. The range of abduction was recorded by measuring the angle formed between the arm axis and parallel to the spinal cord axis. External rotation was measured with the patient standing with the shoulder fully internally rotated and forearm placed transversally over the abdomen. Any rotation from this position was measured and noted as the range of external rotation.

Shoulder and elbow function were graded using the scale proposed by Narakas [2] and Waikakul et al [11] with minimal modification as per Table 1 and Table 2 respectively.

**Table 1:** Grading of shoulder function (Narakas [2] modified):

Grade	Functional status
Poor	No abduction movement and feeling of weightlessness in the limb (MRC 0)
Fair	Stable shoulder without any subluxation but no active movement (MRC I)
Good	Active abduction of < 60 degrees (MRC III) and active external rotation of < 30 degrees
Excellent	Active abduction of > 60 degrees (MRC IV) and active external rotation of > 30 degrees.

**Table 2:** Grading of elbow function (Waikakul et al [11] modified):

Grade	Functional status
Excellent	Ability to lift 2 Kg weight from 0 to 90 degrees of elbow flexion more than 30 times successively.
Good	Ability to lift 2 Kg weight from 0 to 90 degrees of elbow flexion, but less than 30 repetitions successively.
Fair	Motor power more than M3 power but unable to lift a 2 Kg weight.
Poor	Motor power less than M3.

### Surgical technique

All patients were operated under general anesthesia in the supine position with head and trunk turned to the opposite side. The supraclavicular part of the plexus was exposed through a transverse incision 2 cm above the clavicle at the root of the neck and the C5 and C6 roots were identified. Avulsion of the C5–6 roots and absence of usable stump proximally was confirmed intra-operatively. Nerve transfer was performed in the following manner.

### Spinal accessory nerve to suprascapular nerve

**Figure 1:**
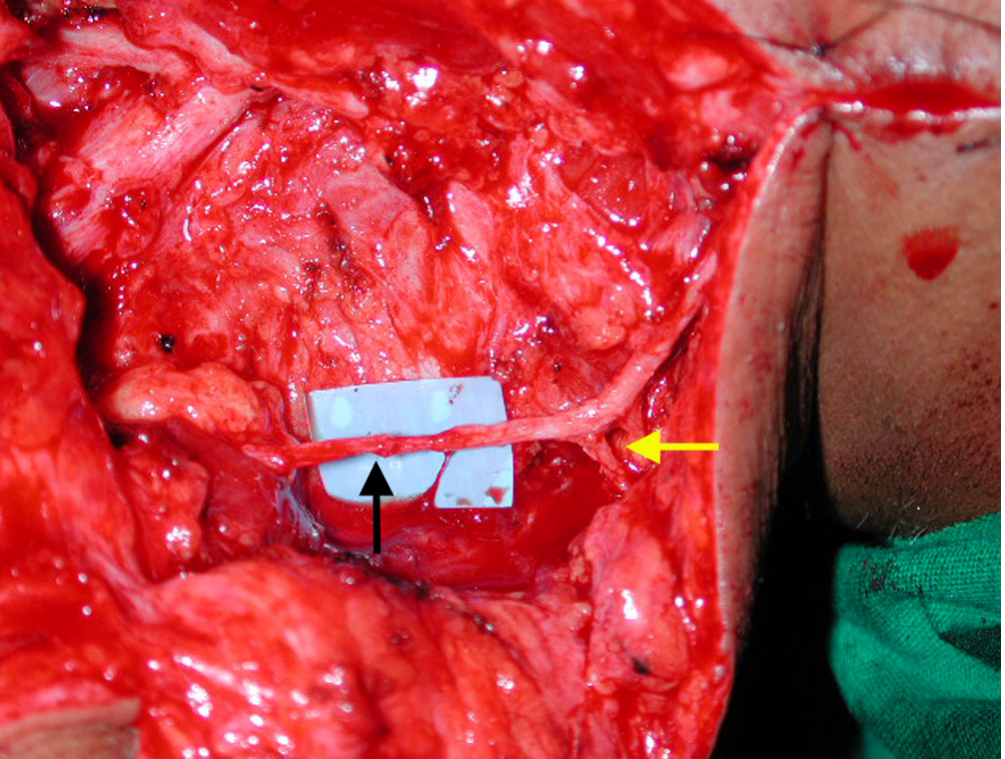
**Spinal accessory to suprascapular nerve transfer**. The proximal branches of the spinal accessory to the upper part of the trapezius are preserved (yellow arrow) and terminal branch is divided and coapted to the suprascapular nerve (black arrow).

The spinal accessory nerve was identified along the superior border of the trapezius and confirmed with electrical stimulation. Suprascapular nerve was then identified as it emerged from the roots in the scar. The proximal branches of the spinal accessory to the upper part of the trapezius were preserved and the terminal branch was dissected and divided as far distally as possible. It was then transposed and coapted to the suprascapular nerve under microscopic magnification with 10-0 Ethilon (Figure 1).

### Oberlin transfer

**Figure 2:**
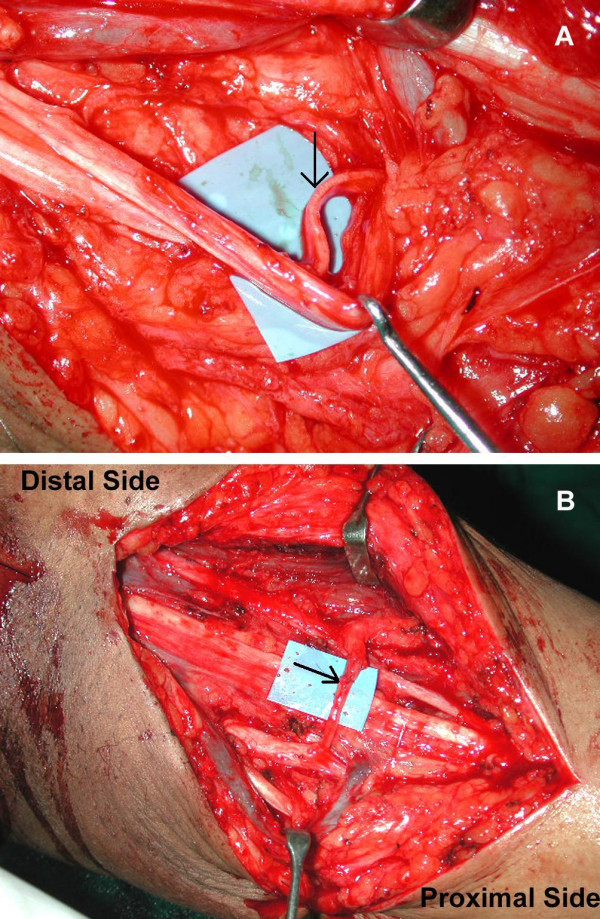
**Oberlin procedure**. The motor fascicles are separated from the rest of the nerve over a distance of 2–3 cm (arrow). The fascicles are turned laterally and superiorly towards the biceps motor branch and coapted with it (arrow).

A 10 cm incision was made over the anteromedial aspect of the arm, starting about 4 cm distal to the pectoralis major lateral border. The musculocutaneous nerve was identified between the biceps and the coracobrachialis muscles. It was traced distally to expose the motor branch to the biceps muscle. The motor branch of the biceps was dissected free and divided for about 2 cm from entry to the muscle. The ulnar nerve was identified through the same incision and confirmed by electrical stimulation. Further dissection was done under microscopic magnification. The epineurium of the ulnar nerve was incised and the fascicles were dissected out. One fascicle of the ulnar nerve was completely isolated and stimulated at low intensity of 0.02–0.04mA to identify the motor fascicles. We insert a piece of glove under this fascicle to completely isolate it from the surrounding fascicles to avoid mass stimulation and false results. It is recommended to take the fascicles in the anterior and medial part of the ulnar nerve which is supposed to contain fibers predominantly to flexor carpi ulnaris. In our experience we found any fascicle of ulnar nerve, irrespective of its anatomical location when stimulated shows contraction in most of the muscles. Hence we choose a fascicle of appropriate size to match the size of the nerve to biceps irrespective of the location in the ulnar nerve. The chosen fascicle is separated from the rest of the nerve. It must be divided 3 cm distal to the level of the possible coaptation to turn laterally to meet the nerve to biceps (Figure 2A). The fascicle is turned laterally and superiorly towards the motor branch of biceps and coapted with 10-0 Ethilon without tension with the ulnar nerve in its usual anatomical position. The wound is closed with a drain placed away from the nerve repair site. The limb is strapped to the chest keeping the shoulder in adducted and internally rotated position and elbow in about 100 degree of flexion for 3 weeks.

### Physiotherapy protocol

Stretching exercises are started at three weeks and electrical stimulation is started after 6 weeks.

## Results

**Figure 3:**
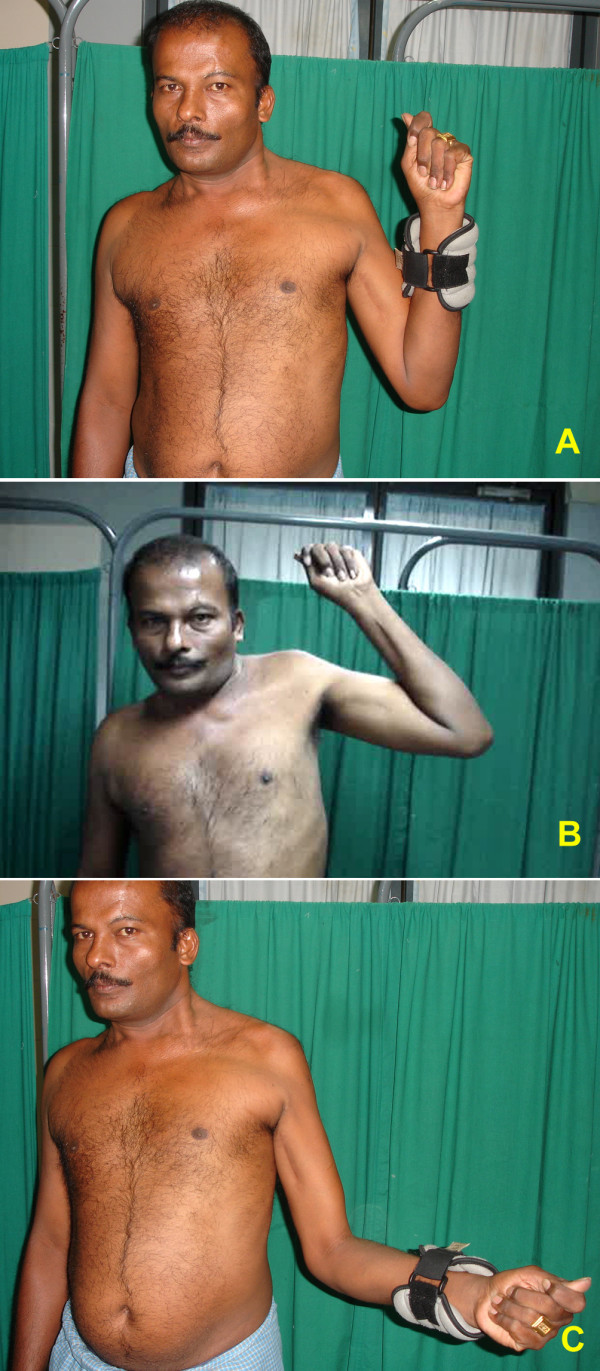
Clinical photographs of case no. 2 showing excellent results for elbow and shoulder function- patient could easily do more than 30 repetitions of elbow flexion with 2 Kg weight and had 95 degree of external rotation and 80 degree of abduction at the shoulder.

**Figure 4:**
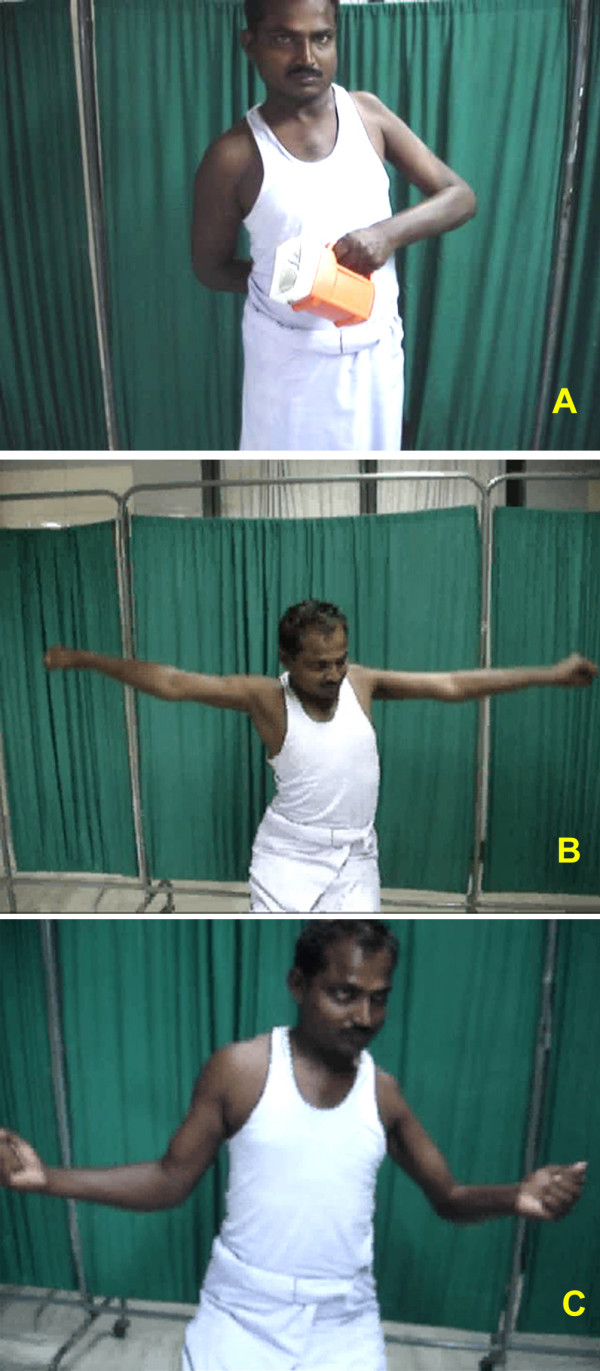
Clinical photographs of case no. 8 showing excellent results for shoulder and elbow function. He had full range of elbow flexion with good power and 90 degree of external rotation and abduction at the shoulder.

All the patients experienced some improvement at their shoulder function. Eight of the 15 patients had recovered active abduction. Five patients had M3 recovery of shoulder abduction and three had M4 recovery. Among the patients who had recovered active movements (eight) average range of abduction was 66 degrees (range 45–90). Figures 3, 4 show a patient with excellent recovery of shoulder abduction. Eight of 15 patients had recovered active external rotation. Among this average external rotation was 44 degrees (range 15–95). The motor recovery of external rotation was M3 in 5 cases and M4 in 3 cases. Figures 3, 4 shows clinical photograph of patient with good recovery of external rotation at the shoulder. 7 never recovered any active abduction or external rotation, but all 7 felt that their shoulder was more stable and developed some control of the limb.

**Figure 5:**
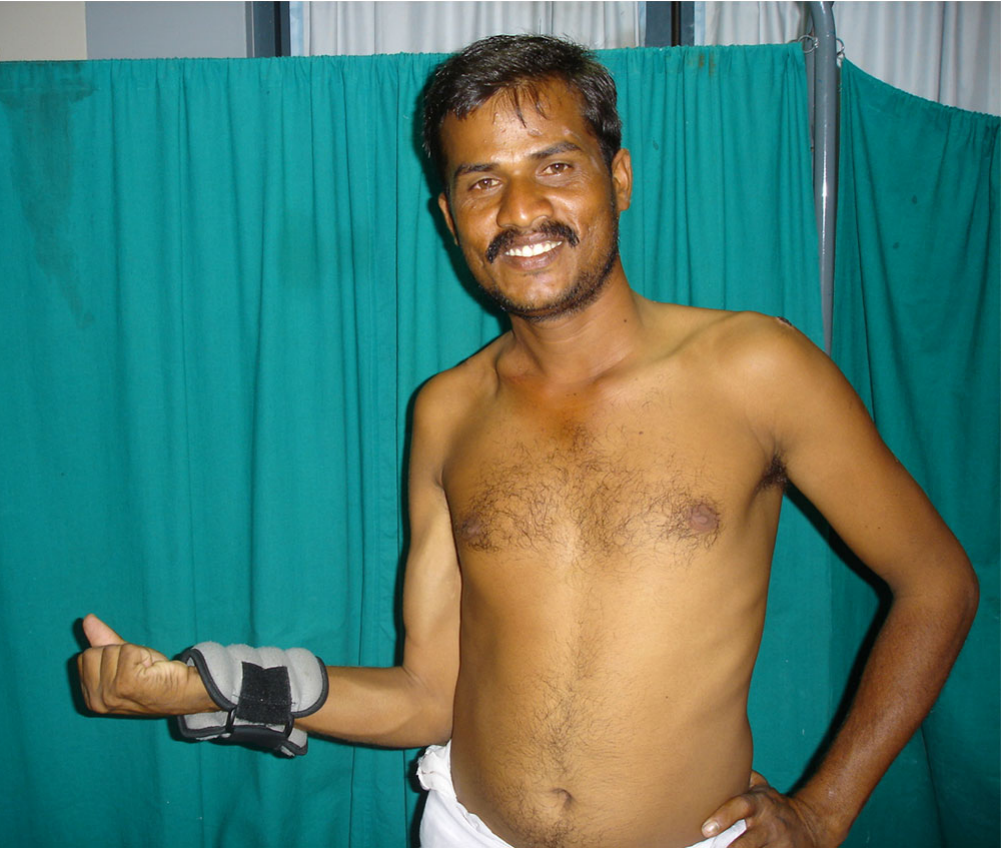
Clinical photograph of case no. 12 showing good results for elbow function but patient had only fair results for shoulder function.

All regained active flexion at the elbow. 13 of 15 patients recovered full flexion (140 degrees); one had 90 degree and other 100 degree of anti-gravity flexion. The average time required for clinical reinnervation of biceps (flicker of movement) was 3 months 9 days (range 1 month 25 days to 4 months). 13 patients had M4 power and 2 had M3+. The average time taken for the recovery of antigravity elbow flexion was 5 months (range 3 1/2 months to 8 months). Figures 3, 4, 5 show clinical photograph of patient with good recovery of flexion at the elbow.

The details of the patients and the final grading of their elbow and shoulder function are detailed in Table 3. Table 4 details the motor grade of the recovered muscles and the final range of movement achieved. Table 5 shows the results with respect to age. Comparable results were achieved in patients below and above 40 years. Table 6 shows the results with respect to the time of surgery since the injury. There was no significant difference between the results in these two groups. Good to excellent results were seen in 13/15 (87%) patients with respect to elbow function but only 8/15 (53%) had good to excellent results for shoulder function.

**Table 3:** Details of the 15 cases in the reported series

No.	Age/Sex	Injury	Procedure	Time since injury (month)	Elbow Flexion grade (Waikakul [11] mod.)	Shoulder Function grade (Narakas [2] mod.)	Follow Up (Months)
1	50/M	C5,6	XI-SSN* + UNF-BrBi**	6	Good	Good	36
2	48/M	C5,6	XI-SSN + UNF-BrBi	3	Excellent	Excellent	34
3	27/M	C5,6	XI-SSN + UNF-BrBi	4	Good	Fair	30
4	42/M	C5,6	XI-SSN + UNF-BrBi	4	Good	Good	26
5	52/M	C5,6,7	XI-SSN + UNF-BrBi	2	Good	Good	25
6	20/M	C5,6,7	XI-SSN + UNF-BrBi	3	Good	Good	23
7	47/M	C5,6	XI-SSN + UNF-BrBi	3	Good	Fair	22
8	45/M	C5,6	XI-SSN + UNF-BrBi	2	Excellent	Excellent	20
9	38/M	C5,6	XI-SSN + UNF-BrBi	4	Fair	Fair	20
10	15/F	C5,6	XI-SSN + UNF-BrBi	5	Good	Fair	18
11	43/M	C5,6	XI-SSN + UNF-BrBi	2	Good	Good	17
12	35/M	C5,6	XI-SSN + UNF-BrBi	4	Good	Fair	15
13	22/M	C5,6	XI-SSN + UNF-BrBi	4	Good	Fair	14
14	24/M	C5,6	XI-SSN + UNF-BrBi	3	Excellent	Excellent	13
15	26/M	C5,6	XI-SSN + UNF-BrBi	3	Fair	Fair	12

*XI-SSN: Spinal accessory to Suprascapular nerve transfer

**UNF-BrBi: Partial nerve transfer of fascicles of ulnar nerve to motor branch of musculocutaneous to biceps muscle.

**Table 4:** Details of the recovery pattern observed.

S. No.	Range of flexion at elbow (degrees) & Motor grading	Range of abduction at shoulder (degrees) & Motor grading	Range of external rotation at shoulder (degrees) & Motor grading
1.	140/M4	60/M3	30/M3
2.	140/M4	80/M4	95/M4
3.	140/M4	0	0
4.	140/M4	60/M3	30/M3
5.	140/M4	50/M3	15/M3
6.	140/M4	45/M3	15/M3
7.	140/M4	0	0
8.	140/M4	90/M4	90/M4
9.	100/M3	0	0
10.	140/M4	0	0
11.	140/M4	50/M3	20/M3
12.	140/M4	0	0
13.	140/M4	0	0
14	140/M4	90/M4	60/M4
15.	90/M3	0	0

**Table 5:** Assessment of results with respect to the injury to surgery interval.

Injury to surgery interval	Excellent/Good results	Fair/Poor results	Total
Less than 3 months	7	1	8
More than 3 months	6	1	7

**Table 6:** Assessment of results with respect to the age of the patients.

Age (yrs)	Excellent/Good results	Fair/Poor results	Total
Less than 40	6	2	8
Greater than 40	7	0	7

## Discussion

The incidence and severity of brachial plexus injury has been increasing world wide mainly because of rapidly growing number of motor cycle accidents. Most of these injuries are high velocity injuries resulting in root avulsions. Although Carlstedl et al [21] have tried repairing roots into the ventral spinal cord and Bertelli & Ghizoni [22] have reported the direct replantation of the nerve graft into the spinal cord with some promising results, the surgical treatment of choice for brachial plexus root avulsion is nerve transfer.

Nerve transfer by reinnervating most functionally important nerves using intact neighboring nerves has become widely accepted since it was reported by Seddon in 1963 [23]. Since then variety of donor nerves have been used for restoring various vital functions. Nerve transfer with various donors has radically improved the prospects for recovery of elbow and shoulder function, especially in patients with irreparable lesion of upper roots. The order of priorities when managing a case of brachial plexus injury is to restore: Full range and power of elbow flexion; shoulder stability; restoration of active abduction and some external rotation. This can be obtained by reinnervation of musculocutaneous and suprascapular nerve. The highest priority in nerve repair is reinnervation of the musculocutaneous nerve to reinnervate biceps [2,13,16]. Biceps also contributes to shoulder stability [13].

Nerve transfer gives good results in restoring simple function like flexion of elbow but results are not as good when done for complex function like shoulder abduction and finger flexion [13]. Allieu et al [3,4], Narakas [1,2] successfully used spinal accessory neurotization for various recipients. Shoulder stability and motion are vital to normal use of the upper extremity. It improves the working space of the hand and makes the hand more useful. Shoulder stability and abduction are frequently accomplished by arthrodesis of shoulder joint. However, according to Narakas [1,2], good suprascapular neurotization will result in twice the range of motion achieved by fusion of shoulder. We agree with Chaung [9] that voluntary control of the shoulder abduction produces more satisfied patients than fusion. Moreover, the reinnervated suprascapular nerve stabilizes the humeral heads and prevents internal rotation of the humerus in patients with reactivated isolated biceps muscle. Merrell et al [10] in their meta-analysis of results of nerve transfer for restoration of shoulder function found that 73% of the patients who underwent nerve transfer for restoration of shoulder abduction achieved M3 power or more but only 26% could achieve M4 or more. The most commonly used donor nerve was spinal accessory (41%). The spinal accessory nerve can be expected to provide a M3 or more strength in about 98% of cases. Also, significantly better results were achieved by reinnervating the suprascapular nerve (92%) than the axillary nerve (69%). They concluded that, shoulder reconstruction should focus on either a spinal accessory nerve to suprascapular nerve transfer or a dual nerve transfer to both suprascapular and axillary nerves. Spinal accessory is suitable for nerve transfer as it is uninjured and lies in close proximity making the transfer easy. It is a large nerve having average of 2000 motor fibers [13], there in no risk of axonal mixing as it is a pure motor nerve, it is present in the same operative field and if divided after the proximal branches to trapezius do not result in significant weakness of trapezius muscle. Reinnervation of suprascapular nerve with spinal accessory has been reported by many authors [1-9]. Shoulder functional recovery after spinal accessory to suprascapular nerve transfer has been modest [10]. The recovery of external rotation is reported to be very less. This can be explained by Narakas hypothesis [24] i.e. the first muscle to be innervated attracts majority of the axons, in this case supraspinatus muscle which is only an abductor attracts all the axons and reduces the potential of reinnervation of the external rotators. The other explanation is, the antagonistic muscle- for instance, the subscapularis muscle- which is needed for humeral head stabilisation remain denervated [7]. Allieu and Cenac [4], Narakas and Hentz [1], Thomeer and Malessy [8] have reported satisfactory function in 0%, 36% and 50% respectively. Chuang et al [9] have proposed dual nerve transfer using simultaneous phrenic nerve to suprascapular nerve and spinal accessory to axillary nerve transfer and achieved shoulder abduction of 20 – 90 degree (mean 55) in all their 21 patients. Using spinal accessory nerve transfer to suprascapular nerve Bertelli et al [7] reported average recovery of 30% of normal range of abduction but there was no external rotation recovery in any of the patients. They found that when only spinal accessory was transferred none of the patients had recovered external rotation. When spinal accessory transfer was supplemented with the transfer of a motor branch of the triceps muscle to teres minor it resulted in good recovery of external rotation with average of 75% of the normal range. They felt that increasing the number of regenerating axons improves the regeneration rate, because in the cases where dual transfer (spinal accessory and long thoracic nerves) to suprascapular nerve was done external rotation was restored. We agree that external rotation is not restored in all the cases but some patients do recover useful external rotation. In our series eight patients had some amount of external rotation (44 degree) and three had more than 60 degree of active external rotation. Our results are much better in contrast to the Bertelli's observation probably because they have selectively applied this transfer to the patients with global brachial plexus palsy but we have used it is patients with upper plexus injury. Suzuki et al [6] have reported long term results of spinal accessory to suprascapular nerve transfer in 12 cases, average abduction of 77.1 degree and external rotation of 16.7 degree was achieved at the shoulder. They also noted that among the patients having functioning serratus anterior muscle 102 degree of abduction and 32.5 degrees of external rotation could be achieved. They suggested that patients with serratus anterior paralysis cannot regain sufficient range of motion by neurotisation of spinal accessory to suprascapular nerve alone because of the consequent instability of scapulothoracic joint. They have recommended repair of long thoracic nerve or stabilization of scapulothoracic joint as part of reconstructive procedure [6]. None of our cases had a parlayed serratus anterior.

Success rate of intercostals to musculocutaneous reported in literature is 33–87% [12]. The success rate depends on the level of the intercostal nerve transaction, the number of nerves anatomosed, and use of nerve graft. El-Gammal & Fathi [12] reported good results in 89.5% probably because three nerves were used and they were directly coapted to the musculocutaneous nerve. Phrenic to musculocutaneous has a reported success rate of about 75% but involuntary movements with respiration and cough persist for about two years. Samardzic et al [13] reported 65% recovery rate with spinal accessory to musculocutaneous nerve transfer and Waikakul et al [11] reported good recovery in 83% of their cases. But since this transfer necessitates use of nerve graft the reinnervation takes long time, in Waikakul's [11] series the electromyographic evidence of reinnervation was first seen at an average of 11.5 months.

In 1994 Oberlin introduced a new technique for restoration of elbow flexion [15]. They transferred about 10% of the fascicles of ulnar nerve to the motor branch of the biceps. Presence of interfascicular connection prevents any deficit in the ulnar nerve distribution following this procedure [15]. Bertelli et al [16], Loy et al [17], Leechavengvongs et al [18] and Sungpet et al [19] have used this method in 10, 18, 32 and 36 cases with consistent good results. In our series good and excellent results were seen in 86.67% which is in accordance with the published studies [16-18]. Loy et al [17] found it to be highly successful in C5–C6 avulsion. They found good results if the procedure is done within months of injury. Antigravity flexion was regularly obtained in less than 6 months without any objective or subjective sequelae of the hand. The greatest advantage of this procedure is early recovery as nerve coaptation is done close to the target muscle without any interposing graft. In our cases the site of coaptation was about 2 cm from the biceps muscle. We observed the clinical evidence of reinnervation as early as 1 month 25 days (average 3 months 9 days). Using a nerve graft always has a great disadvantage as the regeneration of nerve has to cross two barriers. Recent literature [20] and our experience also suggests that technique of double nerve transfer which involves partial transfer of ulna nerve to the biceps motor branch and partial transfer of median nerve to the motor branch to brachialis, gives even better results.

Delay in the surgery is known to result in poor results in brachial plexus surgeries. In our study all the cases were operated within 6 months of injury. When dividing the patient in two groups, less than 3 months and more than 3 months of injury to surgery duration, no significant difference in the results was noted in the present study (Table 5). Although results of nerve surgery are reported to be inversely proportional to the age of the patient, in the present study in the age range of 15 and 52 no significant difference in recovery pattern was noted (Table 6). Functional improvement of arm abduction is better for patients with successful reinnervation of the biceps; eight of our 15 patients who had good or excellent results at shoulder function also had similar improvement at elbow function.

## Conclusion

• Nerve transfer is an effective treatment option for restoration of elbow and shoulder function in brachial plexus injury and multiple nerve transfers help in early restoration of function

• Nerve transfer close to target muscle without any intervening nerve graft allows faster and better recovery.

• Use of ulnar nerve fascicles to restore elbow flexion is reliable technique and the ulnar nerve function is not downgraded.

• Although the functional improvement in shoulder is not as dramatic as elbow, patient satisfaction is phenomenal.

• Age of the patient shall not be criteria to deny the procedure especially till late fifties, and if done within 6 months good results are regularly obtained.

## Competing interests

The authors declare that they have no competing interests.

## Authors' contributions

HR and SRS were the main operating surgeons and designed the study, PB and SRJ performed data collection and analysis of the results, HR and PB were involved in sequence alignment and drafting of the manuscript, SRS edited the manuscript. All the authors have read and final manuscript

## Consent section

Written informed consent was obtained from the patients for publication of this case report and accompanying images. A copy of the written consent is available for review by the Editor-in-Chief of this journal.
